# Mobile home residence as a risk factor for adverse events among children in a mixed rural–urban community: A case for geospatial analysis

**DOI:** 10.1017/cts.2020.34

**Published:** 2020-04-06

**Authors:** Archna A. Patel, Philip H. Wheeler, Chung-Il Wi, Chris Derauf, Euijung Ryu, David Zahrieh, Kara A. Bjur, Young J. Juhn

**Affiliations:** 1 Alix School of Medicine, Mayo Clinic, Rochester, MN, USA; 2 Precision Population Science Lab, Mayo Clinic, Rochester, MN, USA; 3 Department of Pediatric and Adolescent Medicine, Mayo Clinic, Rochester, MN, USA; 4 Child and Family Advocacy Program, Mayo Clinic, Rochester, MN, USA; 5 Department of Health Sciences Research, Mayo Clinic, Rochester, MN, USA; 6 Department of Anesthesiology, Mayo Clinic, Rochester, MN, USA

**Keywords:** Mobile home, adverse events, children, rural, geospatial, hotspot, socioeconomic status

## Abstract

**Background:**

Given the significant health effects, we assessed geospatial patterns of adverse events (AEs), defined as physical or sexual abuse and accidents or poisonings at home, among children in a mixed rural–urban community.

**Methods:**

We conducted a population-based cohort study of children (<18 years) living in Olmsted County, Minnesota, to assess geographic patterns of AEs between April 2004 and March 2009 using International Classification of Diseases, Ninth Revision codes. We identified hotspots by calculating the relative difference between observed and expected case densities accounting for population characteristics (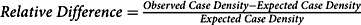; hotspot ≥ 0.33) using kernel density methods. A Bayesian geospatial logistic regression model was used to test for association of subject characteristics (including residential features) with AEs, adjusting for age, sex, and socioeconomic status (SES).

**Results:**

Of the 30,227 eligible children (<18 years), 974 (3.2%) experienced at least one AE. Of the nine total hotspots identified, five were mobile home communities (MHCs). Among non-Hispanic White children (85% of total children), those living in MHCs had higher AE prevalence compared to those outside MHCs, independent of SES (mean posterior odds ratio: 1.80; 95% credible interval: 1.22–2.54). MHC residency in minority children was not associated with higher prevalence of AEs. Of addresses requiring manual correction, 85.5% belonged to mobile homes.

**Conclusions:**

MHC residence is a significant unrecognized risk factor for AEs among non-Hispanic, White children in a mixed rural–urban community. Given plausible outreach difficulty due to address discrepancies, MHC residents might be a geographically underserved population for clinical care and research.

## Introduction

Adverse events (AEs) in childhood continue to represent a significant public health threat to many children in the USA^1^ and are not only reported to be detrimental to physical, mental, and financial health at the time of occurrence but are also associated with an increased rate of asthma, cardiovascular diseases, obesity, alcoholism, diabetes, and depression.^2-4^ They can also result in economic ramifications in the form of lower educational achievement, higher rates of unemployment and low-wage employment, and higher costs of health care for chronic illnesses.^5^ Therefore, it is imperative for new research to better identify children at risk of AEs.

We conceptually defined AEs as both intentional and nonintentional injuries at home, and they include four types of childhood adversity that were identifiable in electronic medical records – two traditional ACEs (physical abuse and sexual abuse) and two additional adversities, home accidents and accidental poisonings. Thus, we used the term “adverse events” to refer to both intentional and unintentional events. Studies assessing childhood adversity have done so primarily within the context of individual and/or familial risk factors, not geographical factors as they tend to focus on associations between adverse childhood experiences (ACEs) and individual socioeconomic status (SES) or proxy measures such as parental education, race, and ethnicity.^4^ Likewise, we recently reported that lower SES – as measured by the individual-level SES measure HOUSES (HOUsing-based SocioEconomic Status) – is an independent risk factor for childhood adversity in the mixed rural–urban community of Olmsted County, Minnesota.^6^

Apart from SES, factors associated with the risk of childhood trauma have been reported relating housing or neighborhood characteristics and a number of different topics including health complaints,^7,8^ cigarette smoking, dental hygiene,^9^ chronic health conditions,^3,10^ functional and cognitive impairment,^11^ inhalation exposures,^8,12-16^ and infection risk. Scant literature addresses the impact of housing and neighborhoods on health outcomes in a well-defined pediatric population with existing studies focusing on the elderly^11^ or high school students.^9^

While the impact of neighborhood environment on other health outcomes has been reported,^17^ at present, little is known about whether AEs occur in a geographically clustered manner, which specific geographic or neighborhood areas are associated with risk of AEs, and what characterizes certain children residing in such areas as high or low risk.

In this respect, geographical hotspot analysis provides a framework for addressing these important study questions with implications for clinical care and public health, as traditional quantitative epidemiological analysis may not capture geospatial patterns for health outcomes. Our study aimed to assess whether AEs occurred in a geographically clustered manner and if our hypothesis was supported to characterize children residing in high-risk geographic areas compared to those residing in low-risk areas. To address these aims, we conducted a population-based retrospective cohort study.

## Methods

### Study Setting and Population

Olmsted County, Minnesota, is a mixed rural–urban Midwestern community located 90 miles southeast of Minneapolis, Minnesota.^18^ In the 2010 US Census, the population of Olmsted County was 83.4% non-Hispanic White, 0.7% Black, 6.4% Asian/Pacific Islander, and 4.2% Hispanic/Latino of any race.^19^ Twenty six percent of the Olmsted County youth population (<18 years of age) were classified as non-Hispanic White.^20^ Seventy-five percent of the population lived in the Rochester urbanized area, 7% in two urban clusters, and 18% in rural areas. Poverty levels in Olmsted County were 8%, well below national (14%) and state levels (11%), with a median family income significantly higher than the national average ($66,252 versus $53,046 from 2009 to 2013).^21,22^ Furthermore, Olmsted County is not a medically underserved area: a large proportion (27%) of residents work in health care, and 95% of adult residents have health insurance.^23,24^ Air quality in Olmsted County, Minnesota, is significantly cleaner than metropolitan cities such as Minneapolis, according to the Minnesota Air Pollution Control Agency.^25^

Olmsted County is an ideal setting to conduct population-based epidemiologic research such as this because 98% of medical care received by county residents is delivered through Mayo Clinic or Olmsted Medical Center and their affiliated health care facilities. The Rochester Epidemiology Project, in operation since 1966, has electronically indexed all inpatient and outpatient episodes of almost all (95%) county residents, including children.^26,27^

The Rochester Epidemiology Project census was utilized to identify all children (age <18 years) who resided in Olmsted County in 2009 and experienced an AE between April 2004 and March 2009, excluding only those individuals without research authorization (<5%). This unique study setting and data source provide a geographically well-defined study population, allowing us to capture nearly all eligible cases and population-based estimates of outcome events. The Institutional Review Boards at Mayo Clinic and Olmsted Medical Center approved this study.

### Case Ascertainment (Adverse Events 2004–2009)

We reported identification methods for children with a history of each AE in a previous study.^6^ Briefly, International Classification of Diseases, Ninth Revision (ICD-9) codes for AEs among children were extracted from the medical records of Olmsted County residents participating in the Rochester Epidemiology Project. These included home accidents (ICD-9 of E849.0), accidental poisonings (E850–858), physical abuse (995.54), and sexual abuse (995.53).^28^ We successfully verified these diagnostic codes for a random sample of 50 from the cohort by conducting medical record review suggesting no misclassification of cases by ICD-9 codes. This present study was an extension of an original, parent study which assessed health disparities in a broad range of health outcomes – including AEs – during the same time interval.^6^

### HOUSES as an Individual-Level SES Measure

The HOUSES index was developed and validated by our research group to assess individual-level SES (not aggregate-level SES), overcoming the unavailability of SES measures in commonly used data sources such as medical records and administrative datasets.^29^ HOUSES is a robust individual-level and objective SES measure represented by a single factor made up of four items (summed *z*-score for number of bedrooms, number of bathrooms, square footage of the unit, and estimated building value of the unit, the higher HOUSES, the higher SES) from publicly available assessment data from the county Assessor’s office. Since its original validation, it has been widely used for clinical and epidemiological studies concerning a broad range of health outcomes and behaviors as well as health care delivery in children and adults.^6,29-43^

### Identifying Areas of Observed and Expected Case Density for AEs

We began by identifying and examining hotspots. In conventional polygon-based analysis, a researcher estimates the number of expected cases in an area (e.g. a Census tract) by applying an overall prevalence proportion to the population of interest. The researcher compares this expected value to the observed value using the relative difference 
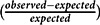
. Our approach followed this model, but instead of using predetermined administrative units, we applied geospatial methods to determine the observed case density and expected case density per square mile as the geographic unit of analysis and calculated 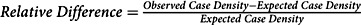.

We estimated observed case density using the kernel density function in ArcMap 10.4.1 (produced by ESRI). This function calculated case density, smoothed according to specified parameters (bandwidth [1 mile] and cell size [330’ × 330’]).^44^ In effect, the method calculates a two-dimensional moving average of case density at each point over the surrounding square mile circle, with point density values averaged for each cell.

We used a one-mile bandwidth because of the following:

1. It had a Moran’s I *z*-score of 3.350 (*p* < 0.001) for all AEs. Larger bandwidths yielded maps with fewer but larger hotspots with less precise relationships to neighborhoods. Narrower bandwidths yielded many more expected case density values close to zero, distorting relative difference calculations, and identified more observed densities with 1 or 2 cases.
2. Urban subdivision activity occurs in increments related to fractions of square miles, and major roads tend to be one mile apart, effectively creating neighborhood boundaries. Land ownership and township roads are also organized on a one-mile grid in rural areas.
3. Olmsted County has both low-density rural areas (11 people/sq. mi.) and high-density urban areas (2000+/sq. mi.). The one-mile bandwidth applied reasonably well to both areas.

We calculated unadjusted expected case density by applying the average prevalence value (3.2%) to each individual’s residential location (i.e. geocoded Rochester Epidemiology Project data points, household address). The sum of each of these values was the expected case density occurring at that location. The residential location was an individual dwelling unit, or in the case of apartment complexes, the parcel. The unadjusted expected case density is based purely on child population. We also calculated expected case density taking into account age/sex-specific prevalence reflecting three age classes (<6, 6–11, 12–18) and SES by applying the HOUSES-specific AE prevalence.

### Relative Difference

**Fig. 1.**
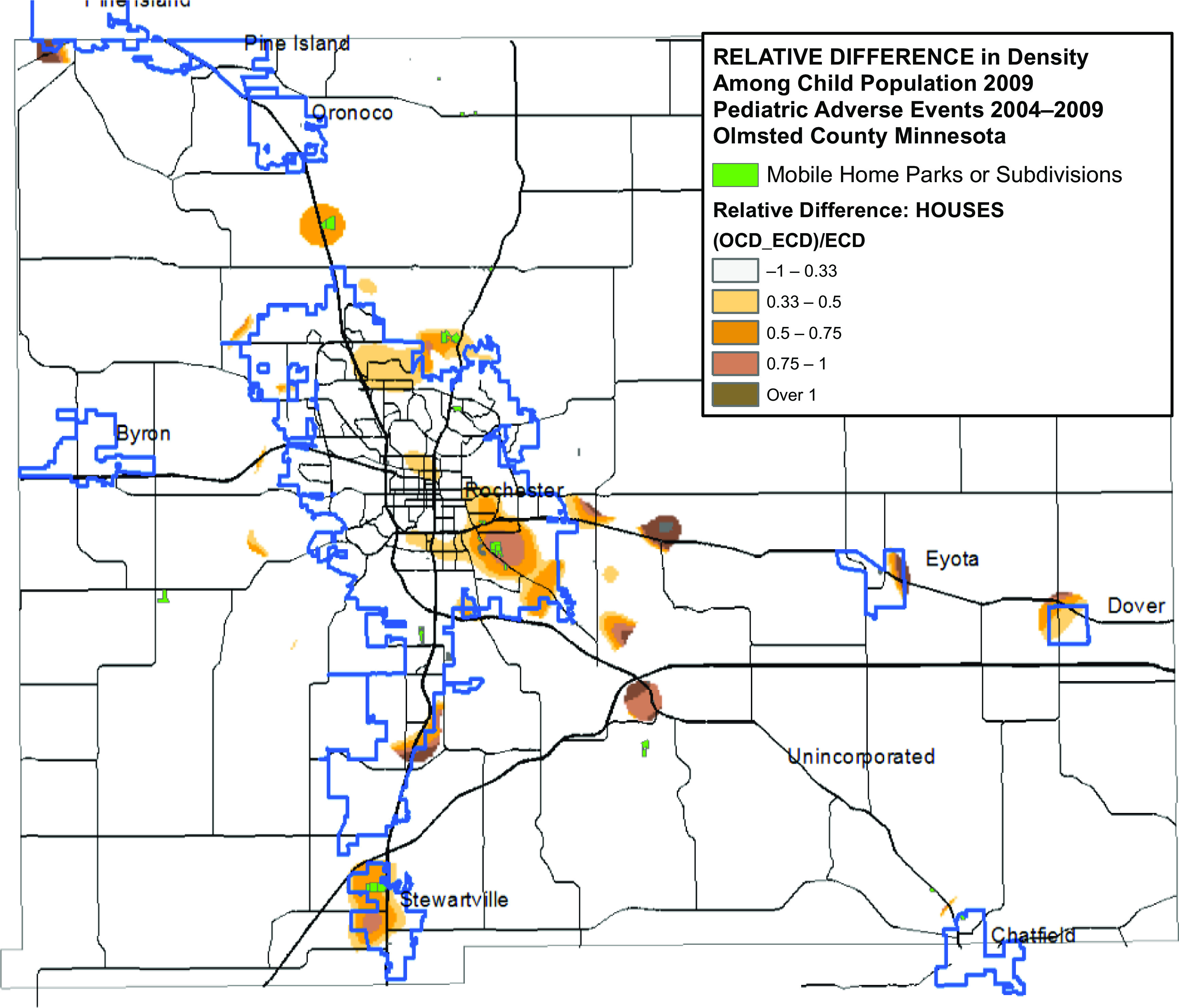
Unadjusted relative difference reflecting child population only.

**Fig. 2.**
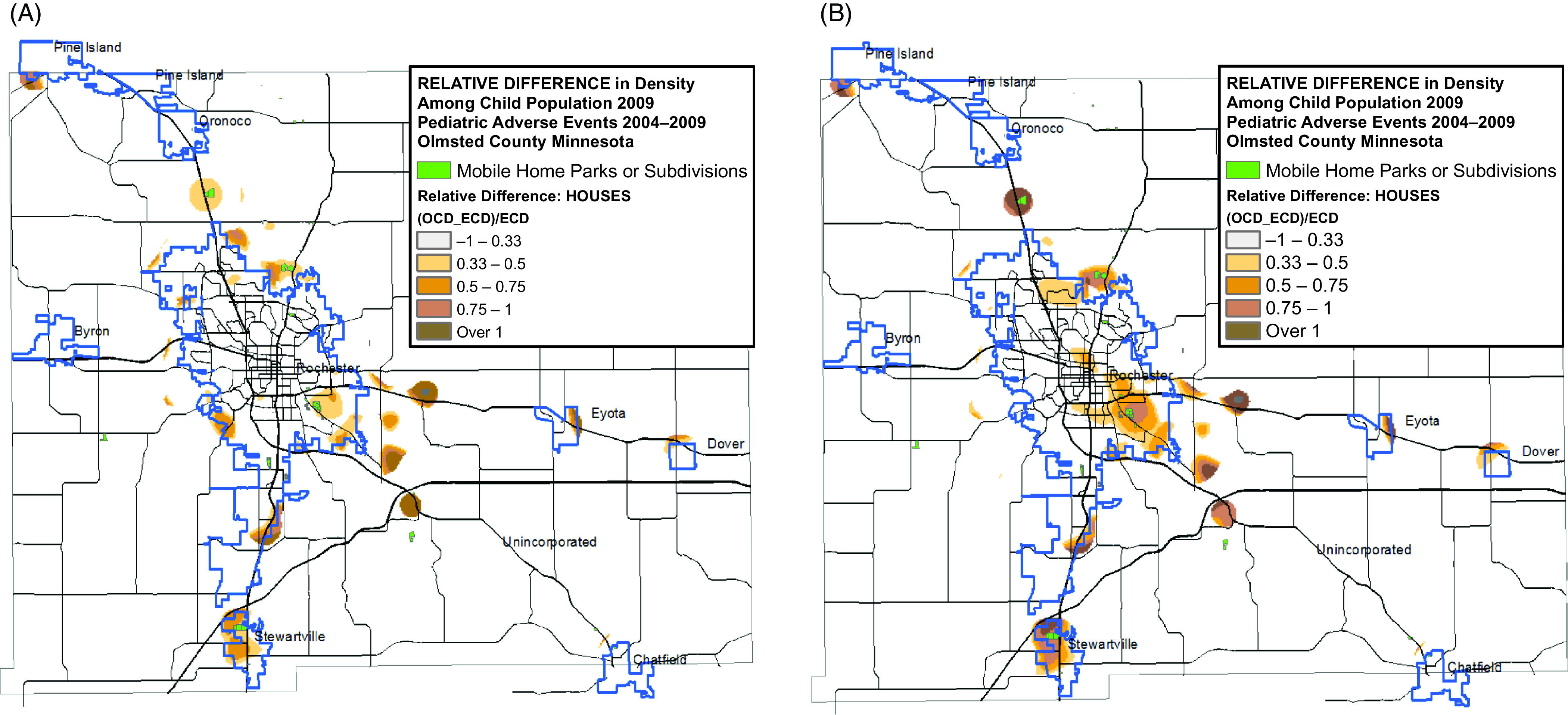
(A) Relative difference of adverse events in childhood adjusted for age/sex. (B) Relative difference of adverse events in childhood adjusted for HOUSES.

We calculated unadjusted (Fig. 1), age/sex-specific, and HOUSES-weighted (Fig. 2) relative differences. A relative difference of 1 indicated that the area had an observed case density value twice the expected value. Relative difference was mapped where the expected case density exceeded a value of 1 per sq. mi. (i.e. where the density of the pediatric population was over 31 children per sq. mi.); a relative difference ≥ 0.33 indicated potential hotspots. Totally, 506 square mile sections had densities below this threshold (total child population 1532, roughly 3 per sq. mi.).

### Statistical Modeling for Assessing Factors Associated with AEs

The hotspot analysis based on kernel density estimation described above was used to visualize geospatial patterns of AE cases, namely, elevated intensity. However, this approach did not provide statistical evidence on whether certain characteristics (e.g. living in a mobile home area) were independently associated with AEs. Thus, we conducted a Bayesian geospatial logistic regression analysis to identify risk factors associated with AEs, accounting for potential observed confounders (e.g. demographic characteristics) and external sources of variation by incorporating spatially correlated heterogeneity effects and family contextual effects. We fit a Bayesian spatial logistic regression model for the binary outcome (AE or no AE), where the probability was a function of a set of explanatory variables, a spatial component, and a family contextual effect (events experienced within the same family shared the same random effect). The child’s residence at April 1, 2009 (when the study cohort was assembled) was used to represent exposure to environmental (e.g. ambient or nonindividual) risk, which was treated as a contextual effect in our model formulation. Conditional on the joint realization of outcomes, we modeled the probability of an AE at any location using integrated nested Laplace approximation (INLA)^45^ to fit the conditional formulation of the spatial model to our population-based data and to obtain posterior quantities of the model parameters. We adopted an intrinsic conditional autoregressive (CAR) prior distribution for spatially correlated heterogeneity.^46^ Following Lawson, the neighborhood relation assumed between-event location was based on a Dirichlet tessellation for a point process where the tiling of the locations leads to sets of natural neighbors defined by adjoining edges of the tile.^47,48^ The family contextual effects were specified as spatially uncorrelated effects and given a normal prior distribution with mean zero and variance σ_γ_2. The prior distribution for each coefficient β attached to a function of an explanatory variable was set to a normal distribution with mean 0 and variance 1000. Lastly, a modestly vague gamma prior (1, 1) was placed on the inverse of the variance components σ_*w*_2 and σ_γ_^2^ associated with spatially correlated heterogeneity and family contextual effects. We fit several Bayesian geospatial logistic regression models, which included all possible two-way interaction terms and higher-order terms of explanatory variables. Explanatory variables considered in the model building process were individual-level covariates: race (non-Hispanic White; other), sex (male; female), HOUSES quartiles, mobile home community (yes; no), and age (in years). The deviance information criterion (DIC >2) was used to compare models.^49^

## Results

### Basic Characteristics of Study Subjects and AE Prevalence

Among the eligible 30,283 children (<18 years), 30,227 (99%) subjects’ residential addresses were successfully geocoded (Table 1). The median age of children included in the study was 8.1 years (25–75th percentile: 3.9–13) with 48.9% females, 85.4% non-Hispanic White, 3.5% Hispanic/Latino, 6.3% Black, and 4.8% Asian/Pacific Islander. Among mobile home community (MHC) residents (*n* = 955), 66.7% were non-Hispanic White and 33.3% were of other minorities (Table 2). Of the study cohort, 974 (3.2%) experienced at least one AE, with 847 (2.8%) being home accidents, 82 (0.3%) poisonings, 21 (0.07%) physical abuse, and 44 (0.15%) sexual abuse. Higher AE prevalence was associated with younger age, minority status, lower HOUSES quartiles (namely, Q1), and MHC residence for any AE (all *p* < 0.001; Table 1). A total of 955 (3.15%) children living in MHCs were identified through the Rochester Epidemiology Project. Of these, 58 (6.1%) children in MHCs experienced AEs compared to 1.7% of children in mobile homes outside MHCs (all of which are in rural areas) (Table 1). The median (IQR) HOUSES *z*-score was 0.17 (−2.01, 2.65). Within MHCs, the median HOUSES *z*-score was −4.97, while the median HOUSES *z*-score outside MHCs was 0.26, suggesting children residing in MHCs had significantly lower SES compared to those residing outside MHCs. The proportion of subjects living in an MHC decreased as the HOUSES index (represented as quartiles) increased (Q1: 96.34%, Q2: 2.72%, Q3: 0.94%, Q4: 0.00%; Cochran-Armitage test for trend *p* < 0.001). Children living in rural MHCs had a higher AE prevalence proportion compared to urban MHCs (7.2% in rural and 5.9% in urban MHCs; data not shown). As shown in Table 2, non-Hispanic White children who resided in MHCs had a higher AE prevalence compared to those outside MHCs. AE prevalence was lower for each minority group in MHCs compared to the same minority group outside MHCs.

**Table 1.** Sociodemographic characteristics of study subjects stratified by adverse event status (at least one event versus none)

Factor	Total (% of total)	Outcome		
		Number of children experiencing any adverse event (% of row)	Number of children experiencing no adverse event (% of row)	*P*^†^
*n* (age <18 years)	30227^*^	974 (3.2)	29253 (96.8)	
Sex				
Female	14795 (48.9)	456 (3.1)	14339 (96.9)	0.19
Male	15432 (51.1)	518 (3.4)	14914 (96.6)	
Age (years)				
<6	11487 (38.0)	404 (3.5)	11083 (96.5)	<0.001
6–11	9631 (31.9)	344 (3.6)	9287 (96.4)	
12–18	9106 (30.1)	226 (2.5)	8880 (97.5)	
Race/ethnicity				
Hispanic/Latino	1061 (3.5)	63 (5.9)	998 (94.1)	<0.001
non-Hispanic White	25806 (85.4)	792 (3.1)	25014 (96.9)	
Black	1895 (6.3)	85 (4.5)	1810 (95.5)	
Asian/Pacific Islander	1465 (4.8)	34 (2.3)	1431 (97.7)	
HOUSES				
Q1	7029 (23.3)	323 (4.6)	6706 (95.4)	<0.001
Q2	6596 (21.8)	231 (3.5)	6365 (96.5)	
Q3	7683 (25.4)	229 (3.0)	7454 (97.0)	
Q4	8919 (29.5)	191 (2.1)	8728 (97.9)	

Adverse events are defined as physical or sexual abuse and accidents or poisonings at home among children in Olmsted County, Minnesota, a mixed rural–urban community, that occurred during the time period April 2004 through March 2009.

HOUSES: HOUsing-based SocioEconomic Status; Q: quartile.

* Total number of study subjects with a geocoded address.

† Based on the chi-squared test, which tested the null hypothesis of no association between each factor and the binary outcome (any adverse event; no adverse event).

**Table 2.** Relationship between mobile home community and any adverse events across race/ethnicity categories

Race/ethnicity category	Lived in mobile home community	Any adverse event		Total	Percent Yes (%)	Odds ratio
		Yes	No			
Non-Hispanic White	Yes	45	592	637	7.1	2.49
	No	747	24422	25169	3.0	
Other^*^	Yes	13	305	318	4.1	0.99
	No	169	3934	4103	4.1	
Total	Yes	58	897	955	6.1	2.00
	No	916	28356	29272	3.1	

Adverse events are defined as physical or sexual abuse and accidents or poisonings at home among children in Olmsted County, Minnesota, a mixed rural–urban community, that occurred during the time period April 2004 through March 2009.

* The other race/ethnic category includes Hispanic/Latino, Black, and Asian/Pacific Islander.

### Identification of Geographical Hotspots with Higher AE Prevalence

Of the nine hotspots, five identified in the geospatial analysis (Fig. 1) overlapped with 11 MHCs in Olmsted County, suggesting neighborhood type (MHCs versus non-MHCs) may affect AE risk. A comparison of population-based unadjusted relative difference, age/sex adjusted relative difference, and HOUSES-adjusted relative difference maps revealed that the hotspots in the vicinity of MHCs persisted despite adjusting for SES and age/sex (Fig. 2).

### Geospatial Analysis Testing Association of MHC with AE Prevalence

The Bayesian geospatial analysis showed that MHC residence was significantly associated with AE prevalence, adjusting for age, sex, SES as measured by HOUSES, and spatial correlation; the association was modified by race/ethnicity. The estimated posterior quantities obtained from the final and most parsimonious model are shown in Table 3. The mean posterior odds ratio for living in an MHC versus not living in an MHC among White children was 1.80 (95% credible interval (1.22, 2.54)). This was not the case for minority children. The mean (95% credible interval) posterior odds ratio for living in an MHC versus not living in an MHC among minority children was 0.82 (0.41, 1.42). Additionally, the effect of age on AE prevalence was modified by HOUSES, such that the protective effect associated with increased age and AEs became more marked with increases in the HOUSES quartiles.

**Table 3.** Estimated posterior quantities from fitting the final Bayesian spatial logistic model

Description of explanatory variable	Parameter	Mean (SD)	95% credible interval
Intercept		−3.3833 (0.1356)	(−3.6538, −3.1212)
Individual-level covariates			
Race (White or other)		−0.1544 (0.0932)	(−0.3351, 0.0305)
Sex (male or female)		0.0852 (0.0659)	(−0.0442, 0.2146)
Housing quartiles		−0.0466 (0.0594)	(−0.1635, 0.0698)
Mobile home community (yes or no)		−0.2448 (0.3210)	(−0.9061, 0.3547)
Age (years)		0.0117 (0.0098)	(−0.0076, 0.0309)
Interaction: mobile home community, race		0.8151 (0.3460)	(0.1603, 1.5198)
Interaction: housing index, age		−0.0159 (0.0057)	(−0.0272, −0.0046)
Random effects			
Spatial component		4.477 (1.397)	(2.351, 7.774)
Family contextual effect		3.169 (1.015)	(1.663, 5.608)

Note: The Bayesian spatial logistic model modeled the binary outcome (any adverse event or no adverse event), where the probability was a function of a set of explanatory variables, a spatial component, and a family contextual effect. Adverse events were defined as physical or sexual abuse and accidents or poisonings at home among children in Olmsted County, Minnesota, a mixed rural–urban community, that occurred during the time period April 2004 through March 2009. Estimates of posterior quantities were obtained from the INLA package. The deviance information criterion was 8469.72. Precisions are presented for the random effects. The corresponding mean (95% credible interval) of the estimated posterior distribution for σ_w_ and σ_γ_ were 0.4892 (0.3590, 0.6502) and 0.5822 (0.4233, 0.7740), respectively.

### Correction of Inaccurate Addresses for Geocoding Process

To improve our geocoding rate, we manually reviewed 2851 addresses (9.4% of total addresses) that were initially nongeocoded before the analysis. Simple typographical errors were updated by referencing an Olmsted County real property data search engine.^50^ If the address was not identified from the county website, we used the subject’s reported addresses within the study period (2004−2009), taking the most recent if multiple were discovered. Totally, 85.5% of mobile home addresses needed review, most of them in MHCs (since there were only 60 isolated mobile homes) compared to 6.8% for all other structure types.

## Discussion

To our knowledge, this is the first study that has demonstrated hotspots of risk for AEs among MHCs.

Our study results showed a significant association between mobile home residence and the risk of AEs among non-Hispanic White children independent of age, sex, and SES; this was not true for minority children. A significant proportion of MHC addresses required manual correction.

Of the 9 hotspots identified by the unadjusted geospatial analysis, 5 overlapped with 11 MHCs (Fig. 1). Accounting for age/sex and SES, as measured by HOUSES, the prevalence of AEs was still higher among children residing in MHCs, compared to that for those residing outside MHCs (Fig. 2). While there is the burgeoning literature which applies geospatial analysis or assesses geographic patterns for health outcomes of interest, in general, geospatial analysis is still underutilized for clinical research.^44,51-54^ Geospatial analysis findings (MHCs as a geographic risk factor for AEs) may not be captured via conventional quantitative analysis, which typically relies on *a priori* list of variables.

After we discovered MHC residence as a risk factor for AE, we estimated the quantitative effect by fitting the data to a Bayesian logistic regression spatial model. Adjusting for other explanatory variables (age, sex, SES) and accounting for the unobserved confounders, the spatial logistic model provided evidence of a novel interaction effect between MHC residence and AE prevalence for non-Hispanic White children (adjusted odds ratio of 1.80 (95% credible interval (1.22, 2.54)). However, this was not true for minorities. Thus, the risk of AEs associated with MHCs may be linked to something *beyond* household SES, age, or sex distribution. It should be noted that while we did not have enough statistical power to sufficiently investigate MHC effect for each of the minority types due to sample size, prevalence of AEs was lower in MHCs than outside MHCs for each minority group. The reasons non-Hispanic, White children residing in MHCs have a higher prevalence of AEs are unknown and need to be elucidated in future studies. Along these lines, it is important to determine the extent to which MHC residence influences unmet health needs beyond AEs (e.g. vaccination, health care access, general checkups, asthma control status, etc.). Our research group is currently addressing this study question.

When addresses in the Rochester Epidemiology Project database were deemed incorrect (i.e. did not exist, did not have the patient living there, contained typographical errors, etc.), they were cross-referenced with local health care providing facilities such as Olmsted Medical Center or Mayo Clinic for correction. The number of addresses needing manual correction was recorded and, as shown in Table 1, 85.5% of addresses that required correction belonged to mobile home residents, mostly in MHCs. It is unclear why a disproportionate amount of addresses were reported inaccurately for MHC residences. We postulate that this may be due to higher frequency of mobility or incomplete reporting or recording of MHC addresses, especially lot numbers. The discovery that mobile home addresses may not be reliable is an important finding because it significantly affects public health interventions which attempt to reach out to residents via postal mail. Integrating special populations into clinical and translational research is a priority of the CTSA program, and MHC residents may currently be significantly underrepresented in this research endeavor.

Our study findings have several implications for clinical care and research. *First*, children in MHCs may be a geographically underserved population at high risk for AEs, inadequately recognized in the literature, and by public health agencies. Clinical care teams and public health agencies at local and national levels should develop interventions in dialogue with residents of MHCs and community-level stakeholders.^1^ *Second*, our observation poses an important question: whether and the degree to which MHC residence impacts other health needs such as preventive care for both children and their families. *Third*, a large number of MHC residents have inaccurate addresses in our health care system. This discrepancy may pose a major challenge for traditional outreach approaches such as mail correspondence (e.g. test results, health education, administrative information, events). *Fourth*, despite the relatively favorable community characteristics (higher family income and insurance coverage (i.e. not an inner-city setting)), significant health disparities still occur in a geographically clustered manner. Health care systems, clinicians, researchers, and policy makers need to be cognizant of this observation. *Lastly*, geographically underserved residents in MHCs may need to be considered a special population as suggested by the National Center for Advancing Translational Sciences because they may be underrepresented in clinical or translational research. MHC residency may also be an important epidemiological factor that needs to be considered for study design and analysis, as it impacts health outcomes and health care access. In this context, geographic hotspot analysis provides a framework in which at-risk subjects can be identified at the population level. Since MHCs are a prevalent neighborhood type in rural and small urban areas, our finding is of particular importance to health providers serving such areas and in targeting efforts to prevent AEs.

Strengths of this study include a population-based design within a self-contained health care environment using the Rochester Epidemiology Project data linkage system which provides a geographically well-defined population and identifies nearly all eligible cases, identification of AEs in childhood based on documented physician diagnoses, and uniform geographical analysis. The use of kernel density techniques to identify geographic concentrations of cases not accounted for by underlying population density, and to test whether suspected factors account for those concentrations, is also advantageous.

This study has the inherent limitations of being a retrospective analysis. Additionally, we presume not all AEs are reported, so these data likely underrepresent true prevalence. A third limitation is data utilization from 2005 to 2009, now roughly 10 years old; however, we strongly suspect that the conclusions remain valid as the characteristics of MHCs in the area have not changed drastically in the last 10 years. Follow-up studies using more recent data and/or looking at different study settings are warranted for policy change and public health intervention. Accidental (home accidents and poisonings) and nonaccidental (physical and sexual abuse) AEs may not share the same risk/protective factors beyond poverty^6^; larger sample sizes may be needed to group them separately and assess if our study findings remain significant for each.

In conclusion, MHC residence is a significant unrecognized risk factor for AEs among non-Hispanic White children. Given address discrepancies making it potentially more difficult to reach out, MHC residents may be a geographically underserved population for clinical care and research. The unique strengths of this study and the novelty of conducting geospatial analysis to recognize at-risk pediatric populations suffering from AEs allow great opportunity for further research and intervention to help prevent future AEs. The study findings are an important addition to the current literature and improve the efficacy of community-based intervention. Directions for further research include replicating our study with different time periods and different geographic areas; addressing other population-level health outcomes; identifying potential characteristics shared by hotspots; or testing intervention strategies.
